# CroR Regulates Expression of *pbp4(5)* to Promote Cephalosporin Resistance in Enterococcus faecalis

**DOI:** 10.1128/mbio.01119-22

**Published:** 2022-08-01

**Authors:** Sarah B. Timmler, Stephanie L. Kellogg, Samantha N. Atkinson, Jaime L. Little, Dušanka Djorić, Christopher J. Kristich

**Affiliations:** a Department of Microbiology and Immunology, Medical College of Wisconsin, Milwaukee, Wisconsin, USA; b Center for Microbiome Research, Medical College of Wisconsin, Milwaukee, Wisconsin, USA; University of Geneva

**Keywords:** two-component signaling system, CroR, Pbp4(5), cephalosporin resistance, *Enterococcus*, Pbp4

## Abstract

Enterococcus faecalis is an opportunistic pathogen and a major cause of severe nosocomial infections. Treatment options against enterococcal infections are declining due to the resistance of enterococci to numerous antibiotics. A key risk factor for developing enterococcal infections is treatment with cephalosporin antibiotics, to which enterococci are intrinsically resistant. For susceptible organisms, cephalosporins inhibit bacterial growth by acylating the active site of penicillin-binding proteins (PBPs), key enzymes that catalyze peptidoglycan cross-linking. Two specific PBPs of enterococci, Pbp4(5) and PbpA(2b), exhibit low reactivity toward cephalosporins, allowing these PBPs to cross-link peptidoglycan in the presence of cephalosporins to drive resistance in enterococci, but the mechanisms by which these PBPs are regulated are poorly understood. The CroS/R two-component signal transduction system (TCS) is also required for cephalosporin resistance. Activation of CroS/R by cephalosporins leads to CroR-dependent changes in gene expression. However, the specific genes regulated by CroS/R that are responsible for cephalosporin resistance remain largely unknown. In this study, we characterized CroR-dependent transcriptome remodeling by RNA-seq, identifying *pbp4(5)* as a CroR regulon member in multiple, diverse lineages of E. faecalis. Through genetic analysis of the *pbp4(5)* and *croR* promoters, we uncovered a CroR-dependent regulatory motif. Mutations in this motif to disrupt CroR-dependent upregulation of *pbp4(5)* in the presence of cell wall stress resulted in a reduction of resistance to cephalosporins in E. faecalis, demonstrating that enhanced production of Pbp4(5) and likely other proteins involved in peptidoglycan biogenesis by the CroS/R system drives enterococcal cephalosporin resistance.

## INTRODUCTION

Enterococci are Gram-positive bacteria and ubiquitous commensals of the gastrointestinal tract of animals and insects (1–3). Over the last few decades, enterococci have emerged as serious opportunistic pathogens, becoming one of the most isolated nosocomial pathogens worldwide (4–6). Enterococcus faecalis and Enterococcus faecium are the most abundant enterococcal species in humans and account for the majority of enterococcal infections (7). These enterococcal infections are increasingly difficult, if not impossible, to treat due to enterococcal intrinsic resistance to numerous antimicrobials and the extraordinary ability of enterococci to hastily acquire resistance to a significant proportion of the antimicrobials put into clinical use (6–9). As a leading cause of life-threatening hospital-acquired infections, multidrug-resistant enterococci have been identified by the CDC as a serious health concern (4, 6, 10–14).

A well-known key predictor for the development of enterococcal infections is prior treatment with cephalosporin antibiotics owing to the intrinsic resistance of enterococci to these antimicrobials (6, 15, 16). Treatment of hospitalized individuals with broad-spectrum cephalosporins leads to alterations of the gut microbiota, which facilitates enterococcal proliferation, followed by translocation of enterococci from the gut into the bloodstream and subsequently into other organs (17). Although the molecular mechanisms contributing to intrinsic cephalosporin resistance in enterococci are not fully known, multiple genetic determinants of this resistance have been identified.

Cephalosporins belong to the beta-lactam family of antibiotics and act by obstructing peptidoglycan biosynthesis through acylation of penicillin-binding proteins (PBPs). The binding of cephalosporins to the active site of PBPs prevents PBPs from cross-linking peptidoglycan, resulting in bacterial cell lysis (18, 19). Two factors required for enterococcal intrinsic resistance to cephalosporins are penicillin-binding proteins Pbp4 (18) and PbpA (20), also known in the literature as Pbp5 (20, 21) and Pbp2b (18), respectively. Pbp4(5) and PbpA(2b) can maintain their cross-linking function in the presence of cephalosporins due to the intrinsically low reactivity of Pbp4(5) and PbpA(2b) toward these antimicrobials (20–22). Currently, it is not well understood how enterococci regulate PBP levels during conditions of cell wall stress to promote resistance to antibiotics.

Another essential determinant of cephalosporin resistance in E. faecalis and E. faecium is the CroS/R two-component signal transduction system (TCS) (23–25). TCSs allow organisms to respond to specific environmental stimuli using a highly conserved phosphoryl relay system in which the sensor-histidine kinase auto-phosphorylates upon recognition of its specific signal, followed by phosphoryl transfer to its cognate response regulator, typically involved in the regulation of gene expression (26). The CroS/R TCS has been shown to play a critical role in enterococcal resistance to multiple cell wall-targeting antimicrobials, including cephalosporins, vancomycin, and bacitracin (23–25, 27–31). The CroS/R TCS consists of two proteins: CroS (transmembrane sensor-histidine kinase) and CroR (OmpR-family response regulator). CroS senses a range of diverse cell wall stressors, as determined by assessing phosphorylation of CroR, and CroR phosphorylation was previously shown to be required for cephalosporin resistance in E. faecalis (24). CroR possesses a functional DNA-binding domain and has been shown to regulate gene expression. Treatment of E. faecalis cells with CroS-activating stimuli results in induction of a CroR-dependent promoter (25, 31).

Prior studies investigating the CroR regulon identified several genes regulated by CroR. In E. faecalis JH2-2, CroR directly regulates the expression of its promoter, those of *salB* (encoding general stress secreted protein), and the *glnQHMP* operon (encoding a predicted glutamine/glutamate transporter) (32, 33). Regions of the *croR* promoter protected by CroR binding have been determined by DNase I footprinting, identifying potential CroR-dependent regulatory motifs (32), although the functional importance of those sequences for CroR-dependent regulation of gene expression was not determined. A more recent study conducted transcriptome analysis using a chimeric response regulator approach to identify CroR-regulated genes in E. faecalis JH2-2, resulting in 50 additional CroR regulon members (27). However, most of the genes identified as CroR regulon members in that study have no known role in enterococcal cephalosporin resistance, so the specific output by which CroS/R contributes to cephalosporin resistance in enterococci has remained unclear.

Prior studies demonstrated differences between E. faecalis JH2-2 and other E. faecalis lineages regarding (i) cell growth and morphology of *croRcroS* deletion strains, (ii) magnitude of cell wall stress resistance phenotypes, and (iii) the identity of CroR regulon members (24, 34). Collectively this work suggested that the CroR regulon as identified in JH2-2 was either incomplete or exhibited diversity in JH2-2 relative to other lineages of E. faecalis. In this study, we performed transcriptome analysis to identify CroR-regulated genes in E. faecalis OG1, revealing dozens of previously unidentified CroR regulon members, including a previously unknown link between CroS/R and Pbp4(5). We discovered a DNA motif required for CroR-dependent regulation of *croR* and *pbp4(5)*. By disrupting CroR-dependent regulation, we determined that Pbp4(5) acted as a downstream effector of CroR, whose upregulation was required for wild-type cephalosporin resistance in multiple E. faecalis lineages.

## RESULTS

### Identification of CroR regulon members.

**FIG 1:**
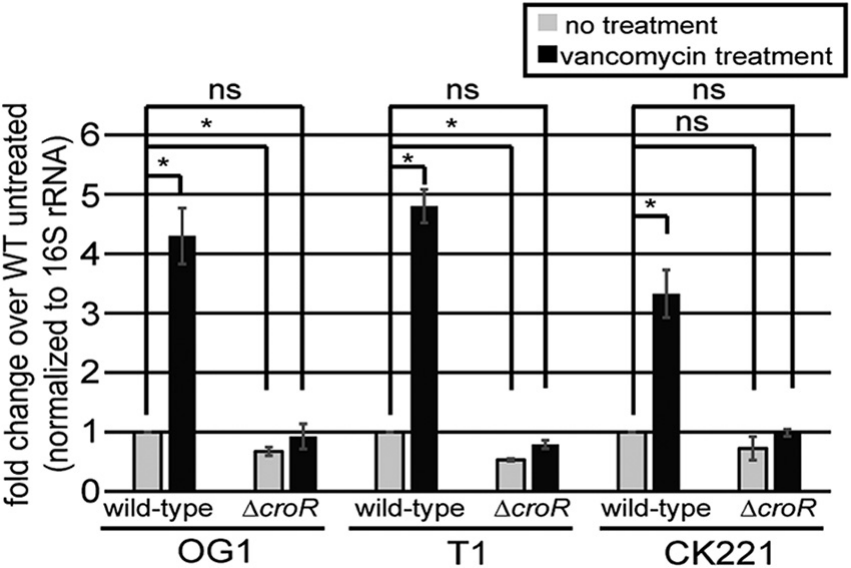
CroR-dependent upregulation of *pbp4(5)* expression upon cell wall stress in multiple E. faecalis lineages. *pbp4(5)* transcript levels from cells grown to exponential-phase then treated (or not) with vancomycin were determined by qRT-PCR. Error bars represent the standard deviation of two independent cultures analyzed in triplicate; ns, not statistically significant (*P* > 0.05) and *, *P* < 0.05, as determined by an unpaired two-tailed parametric *t* test. E. faecalis strains were: OG1 Δ*croR*, SB23; T1 Δ*croR*, SB29; and CK221 Δ*croR*, SB45.

To define the CroR regulon and identify downstream effectors of CroR contributing to cephalosporin resistance in E. faecalis strain OG1, changes in gene expression in wild-type and Δ*croR* strains were assessed by transcriptome sequencing (RNA-seq) with and without exposure to bacitracin, a cell wall acting antibiotic previously shown to robustly activate the CroS/R TCS (24, 25, 31). Tables S1 and S2 list genes that were significantly differentially regulated (+/−1.5-fold; *P* < 0.01) in a CroR-dependent manner. We identified 63 upregulated and 25 downregulated genes from a variety of functional categories, including cell wall synthesis, stress responses, and transcriptional regulators, that largely did not overlap the 50 genes identified in the chimeric response regulator RNA-seq study performed on JH2-2 (27). To validate the RNA-seq data, we performed independent qRT-PCR to analyze the expression of a subset of 21 genes identified via RNA-seq as upregulated in a CroR-dependent manner. For these experiments, RNA was extracted from cells exposed to either bacitracin or vancomycin, which had been previously shown to be a robust activator of the CroS/R TCS (25, 31). All 21 genes tested were induced upon drug exposure in wild-type cells, and induction was eliminated or substantially reduced in Δ*croR* cells (Table S3 and Fig. 1), thereby validating the results of the RNA-seq.

10.1128/mbio.01119-22.5 TABLE S1 List of 63 E. faecalis genes which are significantly (*P* < 0.01) upregulated (+1.5-fold) in a CroR-dependent manner upon CroS/R stimulation in wild-type but not Δ*croR* cells. Genes with predicted involvement in peptidoglycan synthesis are indicated with an asterisk (*). Download Table S1, XLSX file, 0.02 MB. Copyright © 2022 Timmler et al. 2022 Timmler et al. https://creativecommons.org/licenses/by/4.0/ This content is distributed under the terms of the Creative Commons Attribution 4.0 International license.

10.1128/mbio.01119-22.6 TABLE S2 List of 25 E. faecalis genes which are significantly (*P* < 0.01) downregulated (−1.5-fold) in a CroR-dependent manner upon CroS/R stimulation. Download Table S2, XLSX file, 0.01 MB. Copyright © 2022 Timmler et al. 2022 Timmler et al. https://creativecommons.org/licenses/by/4.0/ This content is distributed under the terms of the Creative Commons Attribution 4.0 International license.

10.1128/mbio.01119-22.7 TABLE S3 qRT-PCR validation of RNA-seq data and phenotypic analysis of selected CroR-dependent upregulated E. faecalis genes. Exponentially growing cells of E. faecalis wild-type (OG1) or Δ*croR* mutant (SB23) were exposed (or not) to either bacitracin or vancomycin. qRT-PCR for the indicated gene was performed on extracted RNA, and the fold-change values of treated versus untreated cultures are reported. The column titled “Δ/transposon v WT” reports results from minimal inhibitory concentration (MIC) assays against ceftriaxone (Cx), where “none” indicates no differences for the deletion or transposon mutant in the indicated gene relative to wild-type. The column titled “Δ*croR* with constitutive gene expression” reports results from MIC assays against ceftriaxone for Δ*croR* cells carrying an empty vector or a plasmid with constitutive expression of the indicated gene, where “unchanged” indicates no differences between the two. Genes with predicted involvement in peptidoglycan synthesis are indicated with an asterisk (*). ND, not determined. Download Table S3, XLSX file, 0.01 MB. Copyright © 2022 Timmler et al. 2022 Timmler et al. https://creativecommons.org/licenses/by/4.0/ This content is distributed under the terms of the Creative Commons Attribution 4.0 International license.

It had been previously shown that CroR regulates the expression of the *croR-croS* operon (24, 25, 31, 32). Consistent with these findings, we observed that the *croR-croS* operon (OG1RF_RS12980 and OG1RF_RS12985) was significantly upregulated in a CroR-dependent manner upon exposure to bacitracin (Table S1). Among the other genes significantly upregulated by CroR, eight were predicted or known to be involved in peptidoglycan biosynthesis (Table S1). Because cephalosporins inhibit peptidoglycan biosynthesis, we selected six of these genes for further investigation into their potential role in cephalosporin resistance (Tables 1 and 2, Table S3). Additionally, we observed a significant overlap between the CroR-dependent upregulated genes in our data and those from a previously published transcriptomics study that identified genes upregulated upon antibiotic-mediated cell wall stress (35). We also selected some of these genes to determine if they played a role in cephalosporin resistance (Table S3). To assess these genes for their involvement in cephalosporin resistance, we performed 2 types of experiments: to determine if loss of function mutations (either transposon insertions (36) or in-frame deletions) in the target gene affected ceftriaxone (broad-spectrum cephalosporin) resistance, or if the constitutive expression of chosen upregulated genes enhanced ceftriaxone resistance of the Δ*croR* mutant (Table S3). Of the genes tested, the majority did not alter the ceftriaxone resistance phenotype. However, consistent with a previous report (22), deletion of *pbp4(5)* (OG1RF_RS09755) or *pbpA(2b)* (OG1RF_RS11045) resulted in susceptibility to ceftriaxone. Expression of *pbp4(5)* (25) (Table 2, Table S4), but not *pbpA(2b)* (Table S3 and S4), in the Δ*croR* strain, improved resistance to ceftriaxone (albeit modestly) compared to the Δ*croR* strain (overexpression confirmed by immunoblotting; Fig. S1). Based on these data, the known requirement for Pbp4(5) in cephalosporin resistance, and the CroR-dependent upregulation of Pbp4(5) after exposure to cell wall stress, we selected *pbp4(5)* for further investigation. CroR-dependent upregulation of Pbp4(5) in response to cell wall stress was conserved in a collection of evolutionarily diverse lineages of E. faecalis, including E. faecalis OG1, T1, and CK221 (an erythromycin-sensitive derivative of vancomycin-resistant V583) (Fig. 1), suggesting the underlying genetic circuitry was a representative feature of E. faecalis broadly.

10.1128/mbio.01119-22.1 FIG S1 (A) Pbp4(5) and (B) PbpA(2b) expression analyzed by SDS-PAGE in wild-type (OG1) and Δ*croR* (SB23) strains carrying empty vectors, pJRG9 and pJLL286; or *pbp4(5)* overexpression plasmid, pJLL255, and *pbpA(2b)* nitrate-inducible expression plasmid, pJLL310, grown in the absence or presence of 5mM nitrate (NaNO_3_). The total protein loading control was analyzed using a fluorescent protein labeling reagent (“No-stain”). The same cell lysates were used for immunoblots shown in (A) and (B). Immunoblots are representative of two independent cultures. Download FIG S1, PDF file, 0.3 MB. Copyright © 2022 Timmler et al. 2022 Timmler et al. https://creativecommons.org/licenses/by/4.0/ This content is distributed under the terms of the Creative Commons Attribution 4.0 International license.

10.1128/mbio.01119-22.8 TABLE S4 Ceftriaxone resistance of wild-type or Δ*croR* E. faecalis strains carrying compatible expression plasmids encoding constitutively expressed *pbp4(5)* and/or nitrate-inducible *pbpA(2b)*. Download Table S4, PDF file, 0.2 MB. Copyright © 2022 Timmler et al. 2022 Timmler et al. https://creativecommons.org/licenses/by/4.0/ This content is distributed under the terms of the Creative Commons Attribution 4.0 International license.

### Phosphorylated CroR regulated transcriptional activity of the *pbp4(5)* promoter.

**FIG 2:**
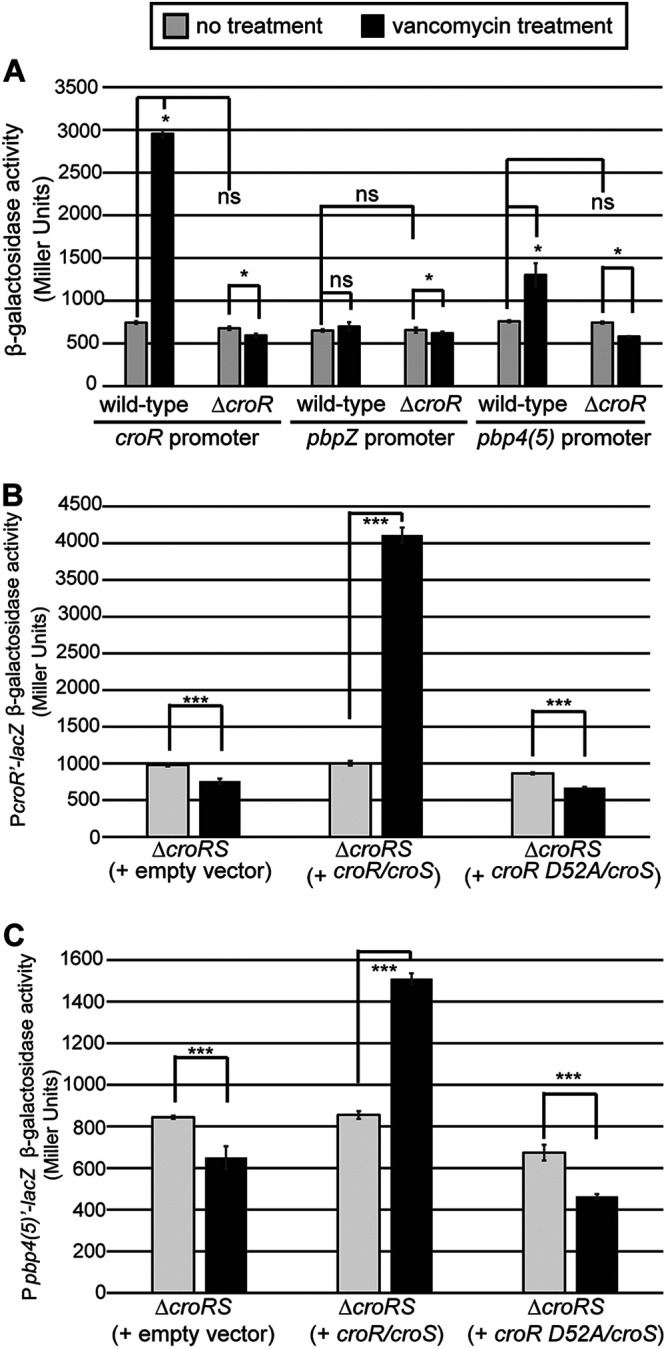
(A) CroR regulates transcriptional activation for *croR* and *pbp4(5)*. The beta-galactosidase activity was determined from *lacZ* fusions to the promoters of *croR* (positive control; pJLL170), *pbpZ* (negative control; pSBT3), and *pbp4(5)* (pSBT8) in exponentially growing OG1 wild-type and Δ*croR* (SB23) cells exposed or not to vancomycin. (B and C) CroR phosphorylation was required for CroR-dependent regulation of *croR* and *pbp4(5)*. Beta-galactosidase activity was determined from *lacZ* fusions to the promoters of (B) *croR* (pJLL170) and (C) *pbp4(5)* (pSBT8) in Δ*croRS* cells (SB35) expressing wild-type *croR/croS* (pJLL59), nonphosphorylatable *croR* D52A/*croS* (pSLB1), or empty vector (pJRG8). Error bars represent the standard deviations from 3 biological replicates; ns, not statistically significant (*P* > 0.05); ***, *P* < 0.0001; *, *P* < 0.05, as determined by an unpaired two-tailed parametric *t* test. In all panels, light gray bars are untreated, black bars are vancomycin-treated.

To test if CroR regulates transcriptional activation of the *pbp4(5)* promoter, we constructed a *lacZ* reporter fusion to the predicted *pbp4(5)* promoter and assessed the resulting β-galactosidase activity. To design *pbp4(5)* promoter fusions for *lacZ* reporter assays, we first identified the transcriptional start site for *pbp4(5)* via the 5′ RACE System (Fig. S2). The +1 position indicates the transcriptional start sites that were determined for *pbp4(5)* and, as a control, for *croR*. The *croR* transcriptional start site identified here corresponded to the previously identified *croR* transcriptional start site (25). Guided by the 5′ RACE System results, we chose a 154-bp fragment, including the first four codons of *pbp4(5)*, encompassing the transcriptional start site and 112 bp upstream. As a negative-control, we constructed a *lacZ* fusion to the predicted promoter of *pbpZ*, encoding a distinct PBP that did not exhibit CroR-dependent changes in gene expression in the RNA-seq data. As a positive-control, we analyzed a previously described *lacZ* fusion to the *croR* promoter (31). β-galactosidase activity was not detected in strains containing the promoter-less *lacZ* construct (unpublished data). In the Δ*croR* mutant, some transcription from the *croR*, *pbpZ*, and *pbp4(5)* promoters was observed, with no statistically significant increases in response to antibiotic-mediated cell wall stress, indicating that some basal transcription was possible without the assistance of CroR (Fig. 2A). As expected (31), we observed elevated β-galactosidase activity from the P*_croR_-lacZ* construct upon treatment with vancomycin to activate CroS/R in the wild-type strain but not in the Δ*croR* strain. Transcriptional activation from the P*_pbpZ_*‘-*lacZ* fusion did not change in wild-type cells treated with vancomycin compared to untreated wild-type cells, consistent with the RNA-seq results. Differences were not detected between OG1 wild-type and Δ*croR* strains. In contrast, we observed enhanced transcriptional activation of *lacZ* from the P*_pbp4(5)_* promoter in the wild-type strain after exposure to vancomycin, and induction was absent in the Δ*croR* strain (Fig. 2A). Altogether, these data demonstrated that transcriptional activation of *pbp4(5)* was regulated in a CroR-dependent manner under conditions of antibiotic-mediated cell wall stress.

10.1128/mbio.01119-22.2 FIG S2 Identification of transcriptional start sites for (A) *croR* and (B) *pbp4(5)* via 5’RACE. The underlined sequence corresponds to previously published *croR* promoter −35 and −10 sequences (TTGTCC-N_18_-TAAAAT) (25). The CroR-dependent regulatory motif identified in this study is shown in bold. N_x_ indicates the number (x) of nucleotides (N) within the bracket sequences. Download FIG S2, PDF file, 0.2 MB. Copyright © 2022 Timmler et al. 2022 Timmler et al. https://creativecommons.org/licenses/by/4.0/ This content is distributed under the terms of the Creative Commons Attribution 4.0 International license.

We previously reported that a CroR variant with a substitution at the predicted phosphoryl-accepting Asp (D52A) does not get phosphorylated *in vivo* and could not drive resistance to ceftriaxone, indicating that CroR phosphorylation was required for CroR function (24). To determine if CroR phosphorylation was required for transcriptional regulation of *croR* and *pbp4(5)*, we performed β-galactosidase assays in Δ(*croR croS)* strains carrying either empty vector, wild-type coexpressed *croR/croS*, or the nonphosphorylatable *croR* D52A/*croS* (Fig. 2B and C). For both P*_croR_*‘- and P*_pbp4(5)_*‘-*lacZ* fusions, we observed an increase in β-galactosidase activity upon exposure to vancomycin in strains expressing wild-type *croR/croS* that did not occur in strains expressing the CroR D52A variant, confirming that CroR phosphorylation was required for CroR to promote transcriptional activation of *croR* and *pbp4(5)* promoters, in accordance with the canonical TCS signaling pathway.

### Identification of a CroR-dependent regulatory DNA sequence motif.

**FIG 3:**
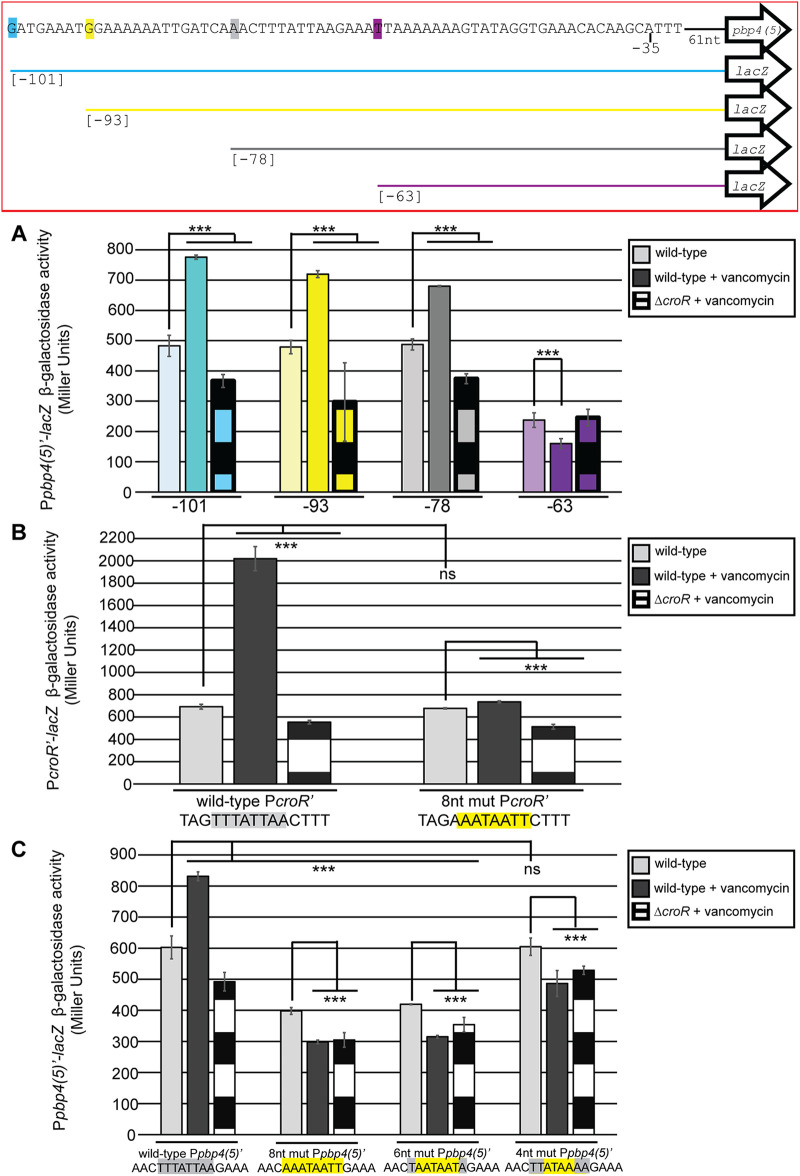
Identification of a CroR-dependent regulatory sequence motif in *croR* and *pbp4(5)* promoters. (A) Beta-galactosidase activity was determined from *lacZ* fusion with promoter truncations for *pbp4(5)*. *pbp4(5)* promoter truncation sequences are shown in the box outlined in red such that the highlighted colored letter indicates the first base of the promoter sequence in each truncation, with the position from the *pbp4(5)* transcriptional start site shown in brackets. *pbp4(5)* promoter truncation sequences beginning at positions −101 nucleotides (blue, pSBT23), −93 nucleotides (yellow, pSBT24), −78 nucleotides (gray, pSBT25), or −63 nucleotides (pink, pSBT26) from the *pbp4(5)* transcriptional start site were assessed. (B) Beta-galactosidase activity was determined from *lacZ* fusion with the promoter of *croR* containing eight nucleotide substitutions, highlighted in yellow, in the identified CroR-dependent DNA regulatory sequence motif. (C) Beta-galactosidase activity was determined from *lacZ* fusion with the promoter of *pbp4(5)* containing varied nucleotide substitutions, highlighted in yellow, in the identified CroR-dependent DNA regulatory sequence motif. Error bars represent the standard deviation of three independent cultures analyzed in triplicate; ns, not statistically significant (*P* > 0.05) and ***, *P* < 0.0001, as determined by an unpaired two-tailed parametric *t* test.

To identify sequences within the *pbp4(5)* promoter that were responsible for CroR-dependent regulation, β-galactosidase assays were performed on a series of progressively shorter *pbp4(5)* promoter truncations (Fig. 3A). We observed CroR-dependent enhanced transcriptional activation upon exposure to vancomycin from all *lacZ* fusions tested except for the shortest truncation beginning at the −63 position (Fig. 3A). These data indicated that CroR-dependent regulation of *pbp4(5)* depended, at least in part, on the 15 nucleotides between positions −78 and −63 (AAACTTTATTAAGAAA). The P*_pbp4(5)_* promoter truncation starting at position −63 resulted in lower overall β-galactosidase activity compared to the other *pbp4(5)* promoter truncations, suggesting that CroR-independent transcriptional regulation was also disrupted to some extent.

Previously published DNase I footprinting assays revealed a 45-bp region of the *croR* promoter that was protected by CroR binding (32). Comparative inspection of the *croR* and *pbp4(5)* promoters identified an eight-nucleotide sequence (TTTATTAA) located within the region of the *croR* promoter protected by CroR binding that was also present within the 15-nucleotide segment of the *pbp4(5)* promoter that was identified in our truncation studies as important for CroR-dependent regulation of *pbp4(5)* (Fig. 3A). The identical eight-nucleotide motif was also present in the *pbp4(5)* promoters of E. faecalis lineages CK221 and T1.

To test if the eight-nucleotide motif was important for CroR-dependent regulation, eight nucleotide substitutions that retained the AT-rich nature of the motif were introduced into the P*_croR_*‘- (Fig. 3B) and P*_pbp4(5)_*‘-*lacZ* fusion plasmids (Fig. 3C) to eliminate the motif. β-galactosidase assays were performed in wild-type and Δ*croR* strains treated or not with vancomycin. Eight-nucleotide (**AAATAATT**) substitutions in the *croR* promoter eliminated CroR-dependent transcriptional activation without altering the basal level of transcription (Fig. 3B), indicating that at least some nucleotides in the TTTATTAA motif were critical for CroR-dependent regulation. However, while the introduction of six- (T**AATAAT**A) and eight- (**AAATAATT**) nucleotide substitutions into the motif of the *pbp4(5)* promoter also eliminated CroR-dependent regulation, those substitutions reduced overall *lacZ* transcription (Fig. 3C). To circumvent this, we introduced four-nucleotide substitutions (TT**ATAA**AA) in the *pbp4(5)* promoter and found that they disrupted CroR-dependent regulation specifically, without altering basal transcription from the P*_pbp4(5)_*‘-*lacZ* fusion (Fig. 3C). Together, these results indicated that at least some nucleotides in the identified sequence motif (TTTATTAA), and presumably those in the central 4 nucleotides of the motif, were essential for CroR-dependent regulation of *croR* and *pbp4(5)*.

### Disruption of CroR-dependent regulation of *pbp4(5)* decreased cephalosporin resistance in E. faecalis.

**FIG 4:**
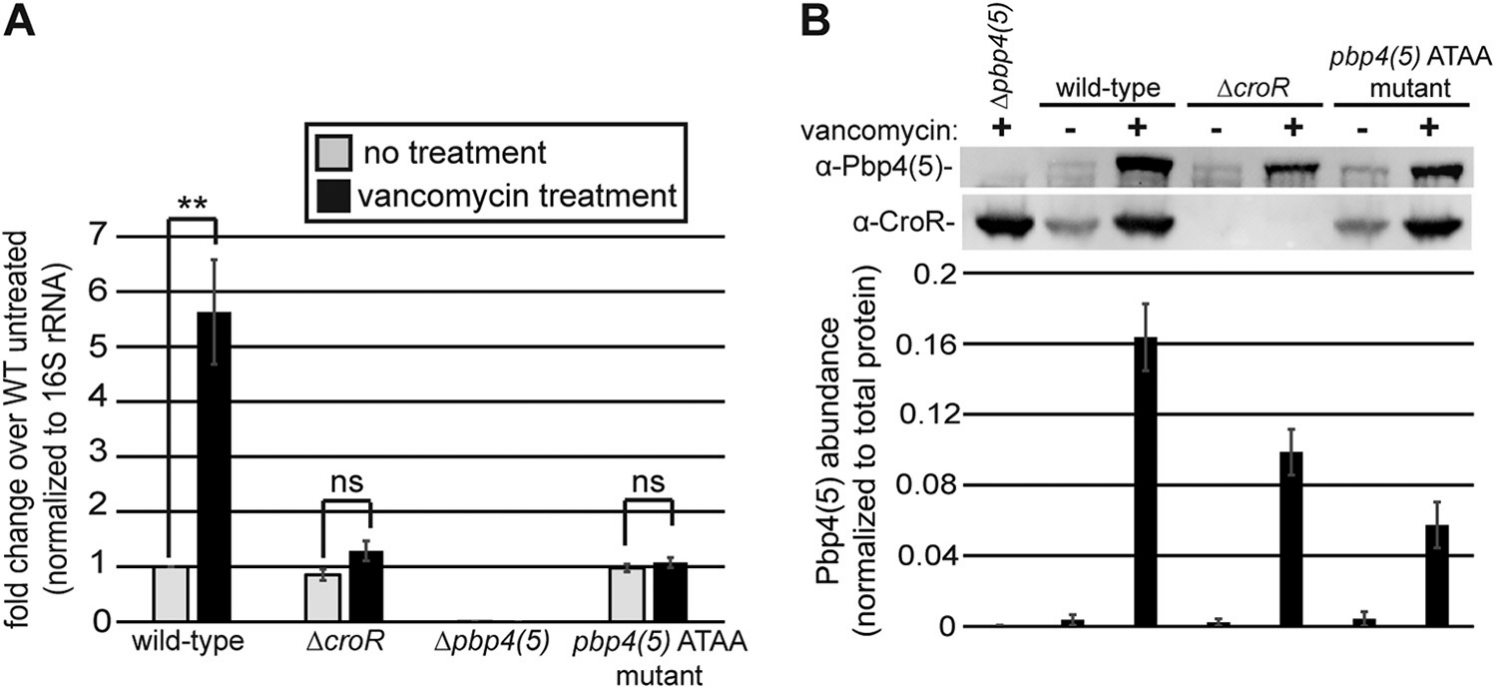
Four nucleotide substitutions within the CroR-dependent regulatory sequence motif in the promoter of *pbp4(5)* disrupts CroR-dependent regulation. Expression of *pbp4(5)* in the absence or presence of CroS/R TCS stimulation (vancomycin) from exponentially growing E. faecalis wild-type (OG1), Δ*croR* (SB23), Δ*pbp4(5)* (JL339), and *pbp4(5)* ATAA (ST8) mutant strains. (A) *pbp4(5)* RNA abundance was analyzed by qRT-PCR. Error bars represent the standard deviation of a minimum of three independent cultures analyzed in triplicate; **, *P* < 0.001, as determined by an unpaired, two-tailed parametric *t* test. (B) Pbp4(5) protein abundance analyzed by immunoblotting; representative of a minimum of three independent cultures. Pbp4(5) abundance was quantified and normalized to total protein. Error bars represent the standard deviation of a minimum of three independent cultures.

To determine if CroR-dependent upregulation of Pbp4(5) contributes to cephalosporin resistance, we introduced the four-nucleotide substitutions from Fig. 3C into the *pbp4(5)* promoter on the chromosome of E. faecalis strains OG1 and CK221 (described here as the *pbp4(5)* ATAA mutants). To confirm that these mutations disrupted CroR-dependent upregulation of *pbp4(5)* from its chromosomal locus, qRT-PCR was conducted using RNA purified from OG1 (Fig. 4A) and CK221 (Fig. S4) wild-type, Δ*croR*, Δ*pbp4(5)*, and *pbp4(5)* ATAA mutant strains treated (or not) with vancomycin to activate CroS/R. Like the Δ*croR* mutant, *pbp4(5)* ATAA mutants exhibited basal levels of *pbp4(5)* expression in the absence of vancomycin similar to the level observed in untreated wild-type cells, but *pbp4(5)* expression did not increase during treatment with vancomycin, demonstrating elimination of CroR-dependent upregulation of *pbp4(5)* in the *pbp4(5)* ATAA mutants (Fig. 4A and Fig. S4). To confirm that disruption of CroR-dependent upregulation of *pbp4(5)* was reflected at the protein level, immunoblotting revealed that the Pbp4(5) protein level in the OG1 *pbp4(5)* ATAA mutant mimicked what was observed in the Δ*croR* strain, confirming the loss of CroR-dependent upregulation (Fig. 4B and Fig. S3A). We also observed a partial increase in the Pbp4(5) protein level in response to vancomycin exposure independently of CroR, which was not observed at the RNA transcript level. The molecular basis for this phenomenon remains unknown (Fig. 4B).

10.1128/mbio.01119-22.3 FIG S3 (A) Total protein signal using fluorescent protein labeling reagent (“No-stain”) as a loading control for Fig. 4B immunoblot. (B) Pbp4(5) expression analyzed by SDS-PAGE in strains carrying the empty vector (1) or *pbp4(5)* overexpression plasmid, pJLL255 (2) with total protein signal shown using fluorescent protein labeling reagent (“No-stain”). Immunoblot representative of two independent cultures. Download FIG S3, PDF file, 0.3 MB. Copyright © 2022 Timmler et al. 2022 Timmler et al. https://creativecommons.org/licenses/by/4.0/ This content is distributed under the terms of the Creative Commons Attribution 4.0 International license.

10.1128/mbio.01119-22.4 FIG S4 Four nucleotide substitutions within the CroR-dependent regulatory sequence motif in the promoter of *pbp4(5)* disrupt CroR-dependent regulation in E. faecalis CK221. Abundance of *pbp4(5)* transcripts from E. faecalis CK221 wild-type, Δ*croR* (SB45), Δ*pbp4(5)* (JL640), and *pbp4(5)* ATAA (ST8) mutant strains grown to exponential-phase with exposure (or not) to 16 μg/mL vancomycin to activate CroS/R. *pbp4(5)* RNA abundance was analyzed by qRT-PCR. Error bars represent the standard deviation of a minimum of two independent cultures analyzed in triplicate; *indicates a *P* value of <0.05, as determined by a two-tailed, unpaired parametric *t*-test. Download FIG S4, PDF file, 0.2 MB. Copyright © 2022 Timmler et al. 2022 Timmler et al. https://creativecommons.org/licenses/by/4.0/ This content is distributed under the terms of the Creative Commons Attribution 4.0 International license.

**FIG 5:**
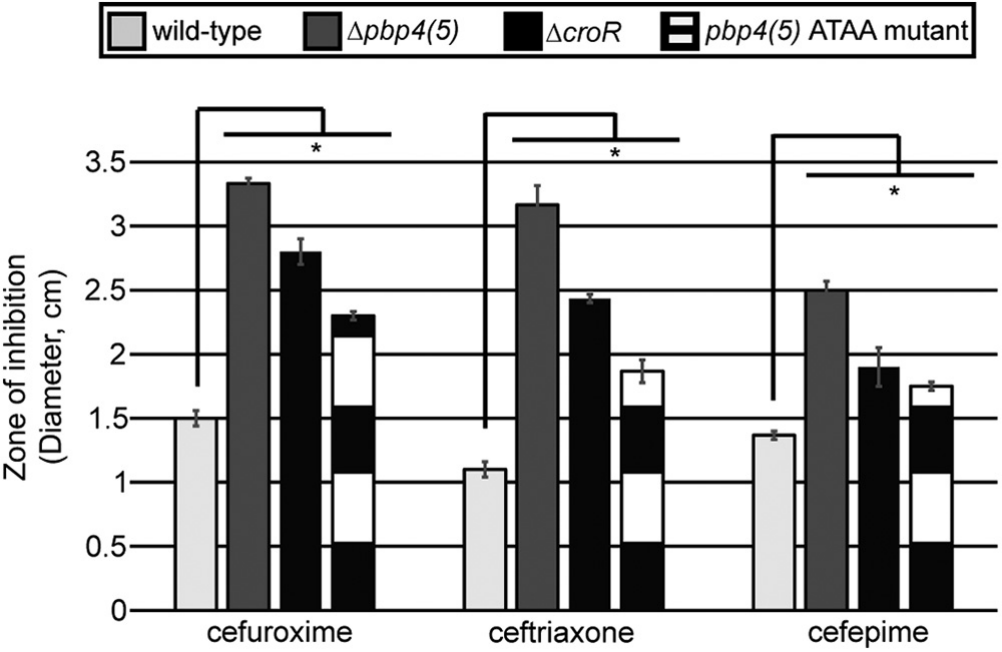
Disruption of CroR-dependent regulation of *pbp4(5)* decreases cephalosporin resistance. Cephalosporin resistance was analyzed by disk diffusion assay. E. faecalis OG1 wild-type, Δ*croR* (SB23), Δ*pbp4(5)* (JL339), and *pbp4(5)* ATAA (ST8) mutant strains were grown in the presence of 6 mm disks loaded with 600 μg cefuroxime (2nd generation), 200 μg ceftriaxone (3rd generation), or 50 μg cefepime (4th generation) cephalosporin antibiotics. Error bars represent the standard deviation of three independent replicates; *, *P* < 0.05, as determined by an unpaired, two-tailed parametric *t* test.

To determine if CroR-dependent upregulation of *pbp4(5)* under conditions of cell wall stress was required for phenotypic cephalosporin resistance in E. faecalis, we performed antimicrobial susceptibility assays for 2nd (expanded spectrum), 3rd (broad spectrum), and 4th generation cephalosporins. *pbp4(5)* ATAA mutants exhibited a reduction in resistance to multiple cephalosporins compared to wild-type strains in both genetic lineages examined, indicating that CroR-dependent upregulation of *pbp4(5)* expression was required for wild-type cephalosporin resistance (Table 1). The cephalosporin resistance levels of the *pbp4(5)* ATAA mutants were higher than the Δ*croR* strains (modestly in most cases), indicating that upregulation of *pbp4(5)* was not the sole CroR-dependent factor that contributes to cephalosporin resistance (Table 1). Overexpression of *pbp4(5)* from a plasmid-borne constitutive promoter in the *pbp4(5)* ATAA mutant (Fig. S3B) resulted in hyper-resistance to ceftriaxone, which was observed for overexpression of *pbp4(5)* in the wild-type and Δ*pbp4(5)* strains (Table 2), confirming that the ATAA mutation within the *pbp4(5)* promoter was responsible for the reduction of cephalosporin resistance in the *pbp4(5)* ATAA mutant. No differences in resistance level were observed between the *pbp4(5)* ATAA mutant and wild-type strain exposed to non-cephalosporin cell wall-targeting antimicrobials vancomycin and ampicillin for both OG1 and CK221 strains (Table 1), which was consistent with the observation that deletion of the *pbp4(5)* gene did not alter resistance to those antimicrobials (Table 1). The results of the MIC experiments were confirmed by disk diffusion assays (Fig. 5). Consistent with the MIC assays, the OG1 *pbp4(5)* ATAA mutant exhibited an intermediate zone of inhibition between OG1 wild-type and Δ*croR* mutant strains (Fig. 5). Collectively, these data illustrated that CroR-dependent upregulation of *pbp4(5)* in response to cephalosporin stress was essential for full cephalosporin resistance in E. faecalis.

**TABLE 1:** Resistance of different E. faecalis strains to antimicrobials

Antibiotic	OG1 MIC (ug/mL)^*a*^				CK221 (erythromycin sensitive V583) MIC (ug/mL)^*a*^			
	Wild-type^*b*^	Δ*croR*	Δ*pbp4(5)*	*pbp4(5)* ATAA mutant	Wild-type^*c*^	Δ*croR*	Δ*pbp4(5)*	*pbp4(5)* ATAA mutant
Cefuroxime (2^nd^ gen)	128	8	≤1	16	512	≤2	≤2	16
Ceftriaxone (3^rd^ gen)	32	4	≤2	16	512	≤4	≤4	16
Cefepime (4^th^ gen)	32	16	4	16	32	4	2	16
Ampicillin	0.5	0.5	0.5	0.5	0.5	0.125	0.5	0.5
Vancomycin	2	0.5	2	2	16	≤4	16	16

a Median MIC determined from ≥2 independent replicates.

b Derivatives of E. faecalis OG1 were Δ*croR*, SB23; Δ*pbp4(5)*, JL339; *pbp4(5)* ATAA mutant (4 bp substitutions in *pbp4(5)* promoter), ST4.

c Derivatives of E. faecalis CK221 were Δ*croR*, SB45; Δ*pbp4(5)*, JL640; *pbp4(5)* ATAA mutant (4 bp substitutions in *pbp4(5)* promoter), ST8.

**TABLE 2:** Ceftriaxone resistance of different E. faecalis strains carrying an empty vector or a plasmid expressing *pbp4(5)*

Strain/plasmid^*a*^	MIC (ug/mL)^*b*^
	Ceftriaxone
Wild-type	
Vector	64
P-*pbp4(5)*	512
Δ*croR*	
Vector	8
P-*pbp4(5)*	16
Δ*pbp4(5)*	
Vector	<2
P-*pbp4(5)*	512
*pbp4(5)* ATAA mutant	
Vector	32
P-*pbp4(5)*	512

a The strains analyzed were as follows: wild-type E. faecalis OG1; Δ*croR*, SB23; Δ*pbp4(5)*, JL339; *pbp4(5)* ATAA mutant (4 bp substitutions in *pbp4(5)* promoter), ST4. The plasmids analyzed were as follows: vector, pJRG9; and *pbp4(5)* overexpression plasmid, pJLL255.

b Median MIC is reported from a minimum of 2 independent replicates.

## DISCUSSION

The CroS/R TCS is known to play a vital role in intrinsic resistance to cephalosporins in enterococci. Though CroR has a functional DNA-binding domain and has previously been shown to regulate gene expression, the specific genes that CroR regulates to promote enterococcal resistance to cephalosporins have been unclear. This study was the first to report that *pbp4(5)* was a member of the CroR regulon and that CroR-dependent upregulation of *pbp4(5)* via the canonical two-component signaling pathway was required for full cephalosporin resistance in E. faecalis.

To define genes of the CroR regulon that mediate cephalosporin resistance, we initially used transcriptomics to identify E. faecalis OG1 genes that are upregulated in a CroR-dependent manner upon antibiotic-mediated cell wall stress. Comparison of the 88 differentially regulated genes found in our study with the 50 genes attributed to the CroR regulon in a recent study of E. faecalis JH2-2 (27) revealed only 3 genes in common: *croR*, *croS*, and serine hydrolase OG1RF_RS02555 (Table S1). The reason(s) for this substantial discordance between the two studies remains unknown. One possibility is that it could reflect significant differences in the experimental setups and environmental conditions used (for example, exposure to antibiotic-mediated cell wall stress in our study that was not part of the experimental design in the JH2-2 study). Alternatively, the discordance might result from genuine differences in the composition of the CroR regulon in the two evolutionarily distinct lineages of E. faecalis (OG1 versus JH2-2 (37)). In a previous study (34), we examined the expression of *salB*, which had been assigned to the CroR regulon in JH2-2 (32), and found that it did not belong to the CroR regulon in OG1 or two other evolutionarily diverse lineages of E. faecalis (T1 and the vancomycin-resistant clinical isolate V583). Consistent with those *salB* results, we found here that CroR-dependent upregulation of *pbp4(5)* was also conserved among the OG1, T1, and V583 lineages, suggesting that the regulon defined in OG1 is likely to be broadly representative of the E. faecalis species.

In support of that hypothesis, a comparison of our transcriptomics results with those of a previous study to define the antibiotic-induced cell wall-stress stimulon of E. faecalis OG1RF (35) revealed that 48 of the 63 CroR- upregulated genes we identified were also members of that cell wall stress stimulon. Hence, it appears that the CroS/R system plays a significant role in global transcriptional remodeling in response to antibiotic-mediated cell wall stress, although other regulators likely also contribute. The importance of CroR-upregulated genes in the response to antibiotic-mediated cell wall stresses is also reinforced by a recent study that used Tn-seq to interrogate the E. faecalis genome, in which CroR itself as well as 5 CroR-upregulated genes from our transcriptomic data set (OG1RF_RS05385, OG1RF_RS11085, OG1RF_RS08985, OG1RF_RS11045 [*pbpA(2b)*] and OG1RF_RS09755 [*pbp4(5)*]) were found to contribute to cell wall-active antimicrobial resistance (28).

Though a previous study dismissed a link between CroR and Pbp4(5), in part because deletion of *croRS* did not alter the basal expression of *pbp4(5)*, that study did not look at *pbp4(5)* levels when enterococci are challenged with cell wall stress (25). Indeed, consistent with those previous results, we observed that some level of *pbp4(5)* expression occurs independently of CroS/R. However, CroR upregulated the expression of *pbp4(5)* in response to cell wall stress, a signaling circuit that was conserved in multiple, diverse E. faecalis lineages (Fig. 1). This CroR-dependent upregulation depends on an AT-rich sequence motif in the *pbp4(5)* promoter (Fig. 3). Introduction of mutations into the AT-rich motif of the *pbp4(5)* promoter eliminated CroR-dependent upregulation of *pbp4(5)* gene expression and concomitantly led to a reduction in cephalosporin resistance in two diverse lineages of E. faecalis, demonstrating that CroR-dependent upregulation of Pbp4(5) was an essential element of the response to cephalosporin stress and integral to the mechanisms of intrinsic cephalosporin resistance (Fig. 4, Fig. S4, and Table 1). However, other CroR-upregulated factors are also required for full phenotypic resistance because loss of Pbp4(5) upregulation does not result in as substantial a defect in resistance as does loss of CroR, and because ectopic expression specifically of Pbp4(5) in the Δ*croR* mutant did not fully rescue cephalosporin resistance. The CroR-dependent transcriptome data contains additional factors involved in peptidoglycan synthesis that likely contribute to cephalosporin resistance in addition to Pbp4(5), including PbpA(2b), a PBP known to be required for cephalosporin resistance; MreC/D, which are membrane proteins that are thought to regulate PBP activity (38–41); the MurT/GatD heterodimer responsible for amidation of peptidoglycan precursors (42–45); and two SEDS-family putative glycosyltransferases, which have been shown to polymerize peptidoglycan in other bacterial species (46–48). It seems likely that CroR-dependent upregulation of some or all of these genes, in particular PbpA(2b), also contributes to wild-type cephalosporin resistance in E. faecalis.

Because DNase I footprinting and EMSAs demonstrated that CroR binds the *croR* promoter, and the *croR* promoter contains the identical AT-rich motif in the CroR-protected region, we suggest that CroR binds directly to this motif in the promoters of its target genes to regulate their expression. Of the six promoters that CroR was recently shown to bind via EMSAs (27), all contained a conserved six-nucleotide motif (TTTATT) corresponding to the first six nucleotides from the AT-rich motif that we identified. In contrast, the motif was absent from promoter sequences that were not bound by CroR, supporting a model in which CroR directly regulates target genes that contain this six-nucleotide motif in their promoters. From our RNA-seq data, 21 of the 63 CroR-dependent upregulated genes contained this six-nucleotide motif (TTTATT) within 200 bp upstream of the predicted gene start codon. This suggests that the CroR regulon is comprised of some genes that are directly regulated by CroR, and others that are indirectly regulated by other CroR-dependent factors. Consistent with this, several putative transcriptional regulators containing the AT-rich motif in their promoters are found within the CroR-upregulated data set and may be responsible for transcriptional regulation of other members of the CroR regulon that lack the AT-rich motif.

In summary, we proposed a model by which CroR controls a complex regulatory network, including *pbp4(5)* and other genes involved in peptidoglycan synthesis, to coordinate a concerted response to antibiotic-mediated cell wall stress. Future work will focus on understanding how downstream effectors of CroR work together to promote resistance to cell wall-targeting antimicrobials. Because the CroS/R TCS is absent from animals and is required for enterococcal resistance to multiple clinically used antibiotics, a better understanding of the mechanisms behind how CroR functions could help identify targets for new antimicrobials.

## MATERIALS AND METHODS

### Bacterial strains, growth media, and chemicals.

Bacterial strains and plasmids used in this study are listed in Table S5. E. faecalis strains were grown in Mueller-Hinton Broth (MHB) (Difco). Escherichia coli strains were grown in lysogeny broth (LB) or half-strength brain heart infusion (BHI) medium (Difco). Erythromycin (Em) was used at 10 μg/mL or 100 μg/mL for E. faecalis and E. coli, respectively. Chloramphenicol (Cm) was used at 10 μg/mL for E. faecalis and E. coli. All cultures were grown aerobically with shaking (225 rpm).

10.1128/mbio.01119-22.9 TABLE S5 Strains and plasmids used in this study. Download Table S5, PDF file, 0.2 MB. Copyright © 2022 Timmler et al. 2022 Timmler et al. https://creativecommons.org/licenses/by/4.0/ This content is distributed under the terms of the Creative Commons Attribution 4.0 International license.

### Plasmid construction.

Plasmids were constructed using Gibson Assembly (49). All inserts in recombinant plasmids were sequenced in their entirety to confirm the absence of mutations. Constructs for *lacZ* fusions with promoter regions for genes of interest were constructed using pSLK234, as previously described (31). Briefly, the region upstream of target genes was amplified by PCR and cloned into pSLK234 generating plasmids listed in Table S5. Plasmids containing substitution variants of the AT-rich motif were generated by introducing mutations into pJLL170 and pSBT25 using Q5 site-directed mutagenesis (NEB).

### Construction of E. faecalis mutants.

**(i) Promoter substitution mutants.** Substitutions were introduced into the *pbp4(5)* promoter on the chromosome of E. faecalis strains OG1 and CK221 to construct mutants using markerless allelic exchange, as previously described (23, 50, 51). Mutant alleles were constructed and introduced into pJH086 with Gibson Assembly. All mutant strains were constructed independently at least twice to ensure the phenotypes were concordant. To verify proper strain construction, PCR was used to amplify the region ±800 bp from the mutation site, and the PCR amplicons were sequenced to confirm the presence of desired mutations.

**(ii) Deletion mutants.** In-frame deletion mutants were constructed in the OG1 strain of E. faecalis using a markerless allelic exchange. Each deletion allele retains codons at the 5′ and 3′ ends of the deleted region (as indicated in Table S5) to avoid perturbing the expression of adjacent genes.

### Antibiotic susceptibility assays.

The MICs of antibiotics were determined as described previously (24). Briefly, bacteria from stationary-phase cultures in MHB (supplemented with 10 μg/mL Cm for plasmid carrying strains) were inoculated at a cell density of ~10^5^ CFU/mL into honeycomb plates containing 2-fold serial dilutions of antibiotic in fresh MHB (supplemented with Cm for plasmid carrying strains). Plates were incubated at 37°C for 24 h in a Bioscreen C plate reader. The optical density at 600 nm (OD_600_) was read every 15 min, with brief shaking before each measurement. MIC values were recorded as the lowest concentration of antibiotics that prevented bacterial growth.

For disk diffusion assays, bacteria from stationary-phase cultures grown overnight in MHB were plated (100 μL) and spread evenly onto MHB agar plates. Disks (6 mm diameter) impregnated with water (negative control), 600 μg cefuroxime, 200 μg ceftriaxone, or 50 μg cefepime were placed in the center, and the plates were incubated overnight at 37°C followed by a measurement of the diameter of the zone of inhibition. A minimum of three independent cultures were analyzed for each E. faecalis strain of interest.

### SDS-PAGE and immunoblotting.

Stationary-phase cultures of E. faecalis strains were diluted in fresh MHB (supplemented with 10 μg/mL Cm for plasmid-containing strains) and grown to exponential-phase at 37°C and 225 rpm (OD_600_ ~0.2). Where specified, vancomycin (3 μg/mL) was added to stimulate the activation of CroR and incubation was continued for 30 min. Bacteria were mixed with an equal volume of cold ethanol-acetone (1:1 vol/vol) mixture to rapidly kill the bacteria and prevent any further physiological changes. Cells were collected by centrifugation, pellets washed with water, and samples normalized to an equivalent OD_600_ before lysozyme treatment (5 mg/mL lysozyme in 10 mM Tris [pH 8], 50 mM NaCl, 20% sucrose) for 30 min at 37°C. 5 × SDS Laemmli sample buffer was added, and samples boiled 5 min before loading on 10% SDS-PAGE gels. Electrophoresis was done using the Laemmli buffer system at room temperature. After electrophoresis, gels were transferred to a polyvinylidene difluoride (PVDF) membrane using a Bio-Rad TurboBlot apparatus (7 min protocol). No-Stain Protein Labeling Reagent (Invitrogen) and corresponding protocol were used to assess total protein on membranes before incubation with anti-Pbp4(5) and anti-CroR custom rabbit polyclonal antisera. Horseradish peroxidase (HRP)-conjugated goat anti-rabbit IgG secondary antibody (Invitrogen) was used for detection on Amersham Typhoon Imager (GE Life Sciences) or BioRad Chemidoc. Azure Spot Software (Azure Biosystems) was used to quantify the total protein and Pbp4(5) signal in each lane. Pbp4(5) abundance was normalized to the total protein signal in each lane when quantifying the Pbp4(5) level to account for any differences in total protein loaded in each lane.

### mRNA extractions for qRT-PCR and 5’ rapid amplification of cDNA ends.

RNA was prepared as previously described (24). Briefly, duplicate or triplicate cultures of E. faecalis wild-type, Δ*croR*, Δ*pbp4(5)*, and *pbp4(5)* ATAA mutant strains were grown in MHB to exponential phase (OD_600_ = 0.2) and treated or not with either 128 μg/mL bacitracin or 3 μg/mL vancomycin (16 μg/mL for strains derived from CK221) for 30 min at 37°C. Cells were mixed with an equal volume of acetone/ethanol (1:1 vol/vol) and collected by centrifugation at 4°C for 15 min at 4,500 rpm. Cell pellets were washed in water and stored at −80°C until extraction. RNA was extracted using a total RNA Minikit (IBI Scientific).

### Analysis of gene expression by quantitative reverse transcription-PCR (qRT-PCR).

DNase Turbo was used to remove any carryover DNA from the above RNA samples, and cDNA was made using SuperScript III first-strand synthesis SuperMix (Invitrogen) per the manufacturer instructions; a no reverse transcriptase control was included in the cDNA synthesis step. A Bio-Rad iCycler and SsoAdvanced SYBR green supermix (Bio-Rad) were used to obtain amplification and melting curves. The primers used are listed in Table S6. Primer efficiencies were determined using serial dilutions of E. faecalis genomic DNA. Calculations of fold changes in gene expression used the Pfaffl method and *16S rRNA* as a reference gene. For gene expression analyses, a minimum of three technical replicates were included for RNA prepared in biological replicates.

10.1128/mbio.01119-22.10 TABLE S6 Primers used in this study. Download Table S6, PDF file, 0.1 MB. Copyright © 2022 Timmler et al. 2022 Timmler et al. https://creativecommons.org/licenses/by/4.0/ This content is distributed under the terms of the Creative Commons Attribution 4.0 International license.

### 5′ rapid amplification of cDNA ends.

We prepared 5′ T-tailed cDNA from total RNA purified as described above. Following manufacturer’s instructions (Sigma-Aldrich), a 5’3′ RACE kit, 2nd generation was used to prepare cDNA from total RNA purified from E. faecalis OG1 and Δ*croR* strains exponentially grown followed by treatment or not with vancomycin. Briefly, first-strand cDNA was made using cDNA synthesis primers *pbp5* SP1 and *croR* SP1 in separate reactions (Table S6). cDNA was purified (Qiagen PCR purification kit) and a homopolymeric A-tail was added to the 3′ ends of first-strand cDNA using recombinant Terminal Transferase and dATP. Using a high-fidelity DNA polymerase, dA-tailed cDNA was amplified using provided Oligo dT-Anchor Primer (and subsequent PCR Anchor Primer) and either primer *pbp5* SP2 (and in a subsequent reaction *pbp5* SP3 primer) and *croR* SP2 (and in a subsequent reaction *croR* SP3 primer) in separate reactions. PCR amplicons were then sequenced to determine the end of the dT tail and the start of the mRNA transcript. There were no differences in transcriptional start site between strains regardless of treatment.

### RNA-seq.

Cultures for each strain and treatment group were prepared using independent biological quadruplicates. Wild-type (OG1) and Δ*croR* mutant (SB23) were grown to exponential-phase in MHB at 37°C and aliquots were treated with bacitracin (128 μg/mL) to activate the CroS/R TCS. After 15 min of treatment (intended to capture the initial transcriptional response to cell wall stress rather than secondary effects of growth inhibition), cultures were harvested by mixing with equal volumes of acetone/ethanol (1:1) as described above. RNA was prepared as described above and subjected to rRNA depletion using the MicrobeExpress kit according to the manufacturer’s instructions (ThermoFisher). Library preparation and sequencing were performed at the University of Minnesota Genomics Center.

### Bioinformatic analysis.

The RNA-seq data described here were submitted to NCBI GEO with accession no. GSE193042.

Raw reads were checked for quality using FastQC v. 0.11.5 (https://github.com/s-andrews/FastQC) trimmed using TrimGalore! v.0.4.4 using default parameters (https://github.com/FelixKrueger/TrimGalore). Bowtie2 v. 2.3.4.3 was used to build a reference to the RefSeq OG1RF reference genome (accession no. NC_017316) (52). Trimmed reads were then mapped back to the reference using Bowtie2, converted to BAM files with Samtools v. 1.3, and the resulting BAM files were sorted (53, 54). HTSeq was then used to count the number of reads per gene using these flags: -s reverse, -t gene, -i Name, and -f bam (55). The resulting count files were then imported into R to run through the DESeq2 pipeline to determine differentially expressed genes (56–58).

### Beta-galactosidase activity assays.

As previously described, CroR-dependent transcriptional activity was monitored using *lacZ* fusion reporter plasmids (Table S5) (31, 51, 59). Stationary-phase cultures of plasmid-bearing strains were diluted to an OD_600_ of 0.01 in MHB supplemented with Cm (and Em for strains carrying pJRG8, pJLL59, or pSLB1) for plasmid selection and grown to exponential phase (OD_600_ ~0.2) at 37°C and 225 rpm. Cultures were split and left unstressed or exposed to 3 μg/mL vancomycin for 30 min before being harvested by centrifugation. Cells were resuspended in Z buffer (60 mM Na_2_HPO_4_-7H_2_O, 40 mM NaH_2_PO_4_-H_2_O, 10 mM KCl, 1 mM MgSO_4_-7H_2_O, 50 mM β-ME), permeabilized with SDS and chloroform for 10 min at RT, and β-galactosidase activity measured using *ortho*-nitrophenyl-β-galactoside (ONPG) (Sigma) as the substrate. Cellular debris was removed by centrifugation and absorbance was measured at 420 and 550 nm; samples were normalized for OD_600_. Samples were analyzed in triplicate, and experiments were performed a minimum of three times.
