# Supplementary material for: CroR Regulates Expression of pbp4(5) to Promote Cephalosporin Resistance in Enterococcus faecalis
Source: mBio. 2022 Aug 1;13(4):e01119-22. doi: 10.1128/mbio.01119-22 (PMC9426447; doi:10.1128/mbio.01119-22)
Supplement: FIG S1 [file mbio.01119-22-s0003.pdf]

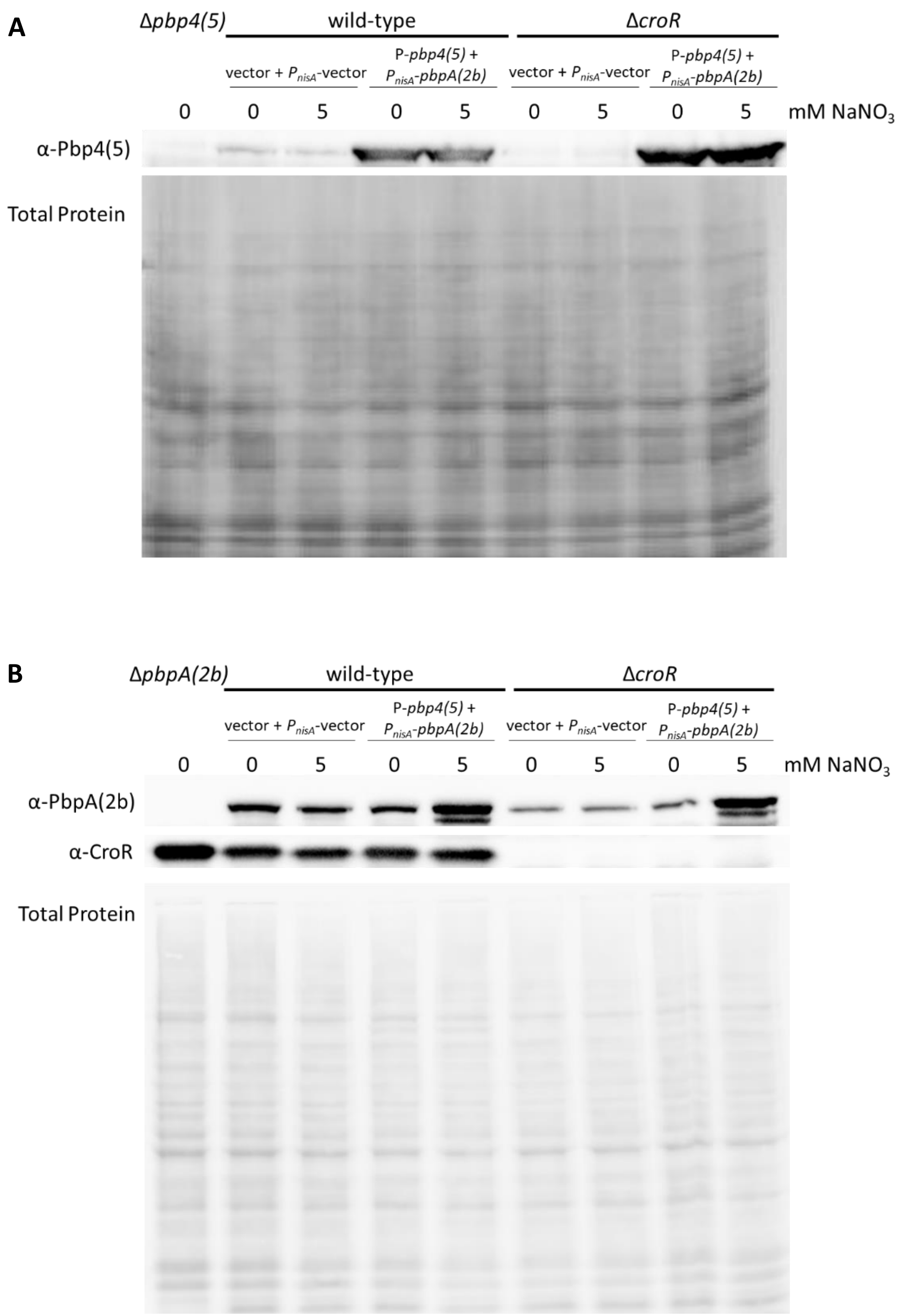

Supplemental Figure 1. (**A**) Pbp4(5) and (**B**) PbpA(2b) expression analyzed by immunoblotting in wild-type (OG1) and Δ*croR* (SB23) strains carrying two compatible expression vectors to enable co-expression of both PBPs. The empty vectors were pJRG9 and pJLL286; a derivative of pJRG9 carrying *pbp4(5)* (pJLL255) enabled constitutive overexpression of Pbp4(5), and a derivative of pJLL286 carrying *pbpA(2b)* (pJLL310) enabled nitrate-inducible expression of PbpA(2b). The concentration of nitrate inducer is indicated. Total protein was analyzed using fluorescent protein labeling reagent

("No-stain"). The same cell lysates were used for immunoblots shown in (A) and (B). Immunoblots are representative of two independent cultures.
