# Supplementary material for: CroR Regulates Expression of pbp4(5) to Promote Cephalosporin Resistance in Enterococcus faecalis
Source: mBio. 2022 Aug 1;13(4):e01119-22. doi: 10.1128/mbio.01119-22 (PMC9426447; doi:10.1128/mbio.01119-22)
Supplement: TABLE S4 [file mbio.01119-22-s0007.pdf]

Supplemental Table 4. Ceftriaxone resistance of wild-type or Δ*croR E. faecalis* strains carrying compatible expression plasmids encoding constitutively expressed *pbp4(5)* and/or nitrate-inducible *pbpA(2b)*.

**Strain/plasmids**[a]	**Ceftriaxone MIC (ug/ml)**[b]	
	**0 mM $NaNO_3$**	**5 mM $NaNO_3$**
wild-type /		
vector + P_{nisA}-vector	64	128
P-*pbp4(5)* + P_{nisA}-vector	512	512
vector + P_{nisA}-*pbpA(2b)*	64	128
P-*pbp4(5)* + P_{nisA}-*pbpA(2b)*	512	512
Δ*croR /*		
vector + P_{nisA}-vector	8	8
P-*pbp4(5)* + P_{nisA}-vector	32	32
vector + P_{nisA}-*pbpA(2b)*	8	8
P-*pbp4(5)* + P_{nisA}-*pbpA(2b)*	32	16

[a]The strains analyzed were as follows: wild-type *E. faecalis* OG1; and Δ*croR*, SB23. The plasmids analyzed were as follows: vector, pJRG9; *pbp4(5)* overexpression plasmid, pJLL255; nitrate-inducible vector, pJLL286; *pbpA(2b)* nitrate-inducible expression plasmid, pJLL310.
[b]Median MIC is reported from a minimum of 2 independent replicates.
