# Supplementary material for: CroR Regulates Expression of pbp4(5) to Promote Cephalosporin Resistance in Enterococcus faecalis
Source: mBio. 2022 Aug 1;13(4):e01119-22. doi: 10.1128/mbio.01119-22 (PMC9426447; doi:10.1128/mbio.01119-22)
Supplement: FIG S2 [file mbio.01119-22-s0004.pdf]

A *croR* promoter sequence

transcriptional start site   translational start site

TGGCTATAG**TTTATTAA**CTTTTAGCAAAATCTTCTAAATTTATCGTAATATATCAAGGATTTTGTCCTTTTTAGCAATTATTTGTTAAAATATGGAAAAGAGATTTTTTTATATATGGAGGAAATGAACGCTTATG

-95 -85 -75 -65 -55 -45 -35 N_{18} -10 +1 N_{38}

B *pbp4(5)* promoter sequence

transcriptional start site   translational start site

GATGAAATGGAAAAAAATTGATCAAAC**TTTATTAA**GAAATTAAAAAAAGTATAGGTGAAACACAAGCATTTTAAAAAAAGTTACGTATAATAGAAGAAAAGACATATTATATAGTTAGGGGATTATTGAATG

-101 -95 -85 -75 -65 -55 -45 -35 -10 +1 N_{30}

Supplemental Figure 2. Identification of transcriptional start sites for (A) *croR* and (B) *pbp4(5)* via 5'RACE. The underlined sequence corresponds to previously published *croR* promoter -35 and -10 sequences (TTGTCC-N_{18}-TAAAAT) (Comenge 2003). The **CroR-dependent regulatory motif** identified in this study is shown in bold. N_x indicates the number (x) of nucleotides (N) within the bracket sequences.
