# Supplementary material for: CroR Regulates Expression of pbp4(5) to Promote Cephalosporin Resistance in Enterococcus faecalis
Source: mBio. 2022 Aug 1;13(4):e01119-22. doi: 10.1128/mbio.01119-22 (PMC9426447; doi:10.1128/mbio.01119-22)
Supplement: FIG S3 [file mbio.01119-22-s0005.pdf]

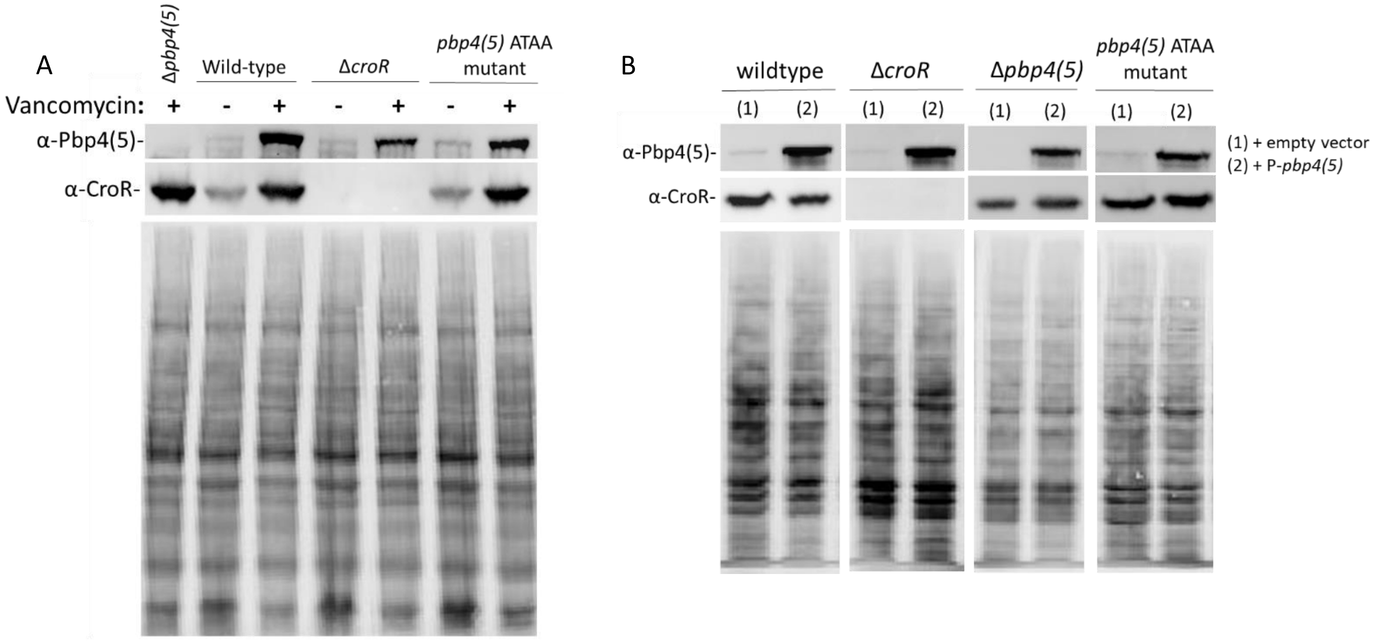

Supplemental Figure 3. (A) Total protein signal using fluorescent protein labeling reagent ("No-stain") as a loading control for Figure 4B immunoblot. (B) Pbp4(5) expression analyzed by immunoblotting in strains carrying empty vector (1) or *pbp4(5)* overexpression plasmid, pJLL255 (2) with total protein signal shown using fluorescent protein labeling reagent ("No-stain"). Immunoblot representative of two independent cultures.
