# Supplementary material for: CroR Regulates Expression of pbp4(5) to Promote Cephalosporin Resistance in Enterococcus faecalis
Source: mBio. 2022 Aug 1;13(4):e01119-22. doi: 10.1128/mbio.01119-22 (PMC9426447; doi:10.1128/mbio.01119-22)
Supplement: FIG S4 [file mbio.01119-22-s0006.pdf]

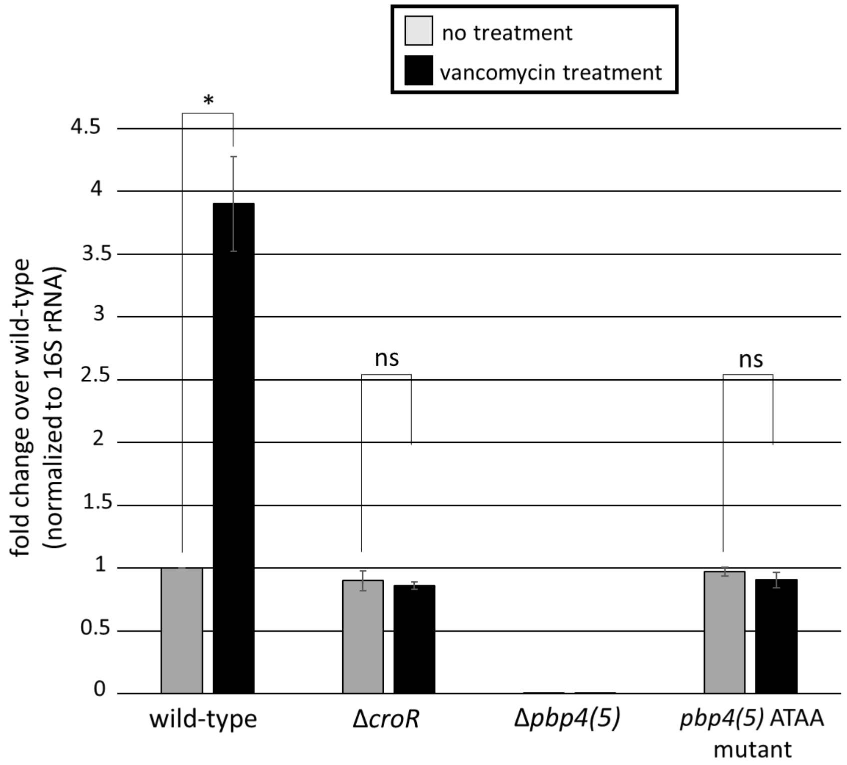

Supplemental Figure 4. Four nucleotide substitutions within the CroR-dependent regulatory sequence motif in the promoter of *pbp4(5)* disrupts CroR-dependent regulation in *E. faecalis* CK221. Abundance of *pbp4(5)* transcripts from *E. faecalis* CK221 wild-type, Δ*croR* (SB45), Δ*pbp4(5)* (JL640), and *pbp4(5)* ATAA (ST8) mutant strains grown to exponential phase with exposure (or not) to 16 µg/ml vancomycin to activate CroS/R. *pbp4(5)* RNA abundance analyzed by RTqPCR. Error bars represent standard deviation of a minimum of two independent cultures analyzed in triplicate; *indicates a P value of <0.05, as determined by a two-tailed, unpaired parametric *t*-test.
