# Supplementary material for: CroR Regulates Expression of pbp4(5) to Promote Cephalosporin Resistance in Enterococcus faecalis
Source: mBio. 2022 Aug 1;13(4):e01119-22. doi: 10.1128/mbio.01119-22 (PMC9426447; doi:10.1128/mbio.01119-22)
Supplement: TABLE S5 [file mbio.01119-22-s0008.pdf]

**Supplemental Table 5**. Strains and plasmids used in this study.

| Strain | Genotype or description | Source or reference |
|---|---|---|
| Strains | | |
| *E. coli* | | |
| Top10 | Routine cloning host | Lab stock |
| DH5α | Routine cloning host | Lab stock |
| | | |
| *E. faecalis* | | |
| OG1 | Wild-type laboratory strain isolate (MLST 1) | 1 |
| SB23 | OG1 Δ*croR* | 2 |
| SB35 | OG1 Δ(*croR croS)* | 3 |
| ST4 | OG1 *pbp4(5)* ATAA promoter mutant (4 nucleotide substitutions: ATAA) | This work |
| JL339 | OG1 Δ*pbp4(5)* | 4 |
| V583 | Vancomycin-resistant clinical isolate (MLST 6) | 5 |
| CK221 | V583 Δ*ermB* (Erm$^{S}$) | 6 |
| SB45 | CK221 Δ*croR* | 7 |
| ST8 | CK221 *pbp4(5)* ATAA promoter mutant (4 nucleotide substitutions: ATAA) | This work |
| JL640 | CK221 Δ*pbp4(5)* | 8 |
| T1 | Wild-type (MLST 21), CDC reference strain | 9 |
| SB29 | T1 Δ*croR* | 6 |
| JL632 | OG1 Δ*pbpA(2b)* | 8 |
| SB77 | OG1 Δ*OG1RF_RS05215* | This work |
| SK129 | OG1 Δ*OG1RF_RS12755* | This work |
| SB93 | OG1 Δ*OG1RF_RS02555* | This work |
| 8N14 | OG1RF *OG1RF_RS03660::Tn* | 10 |
| 13D12 | OG1RF *OG1RF_RS05550::Tn* | 10 |
| 2M10 | OG1RF *OG1RF_RS05565::Tn* | 10 |
| 7B1 | OG1RF *OG1RF_RS05385::Tn* | 10 |
| 25M20 | OG1RF *OG1RF_RS05555::Tn* | 10 |
| | | |
| Plasmids | | |
| pCI3340 | *E. coli-E. faecalis* shuttle vector (Cm$^{r}$) | 11 |
| pSLK234 | Promoterless *lacZ* in pCI3340 | 12 |
| pJLL170 | $P_{croR}$'-*lacZ* in pCI3340 (includes 240bp upstream of the translational start site) | 12 |
| pSBT3 | $P_{pbpZ}$'-*lacZ* in pCI3340 (includes 400bp upstream of the translational start site) | This work |
| pSBT8 | $P_{pbp5}$'-*lacZ* in pCI3340 (includes 154bp upstream of the translational start site) | This work |
| pJRG8 | *E. faecalis* expression vector (Erm$^{r}$) | 13 |
| pJLL59 | $P_{croR}$-*croR croS* in pJRG8 | 3 |
| pSLB1 | $P_{croR}$-*croR* D52A *croS* in pJRG8 | 3 |
| pSBT23 | $P_{pbp5}$'-*lacZ* in pCI3340 (includes 130bp upstream of the translational start site) | This work |
| pSBT24 | $P_{pbp5}$'-*lacZ* in pCI3340 (includes 120bp upstream of the translational start site) | This work |
| pSBT25 | $P_{pbp5}$'-*lacZ* in pCI3340 (includes 105bp upstream of the translational start site) | This work |
| pSBT26 | $P_{pbp5}$'-*lacZ* in pCI3340 (includes 90bp upstream of the translational start site) | This work |
| pSBT29 | pSBT25 with 8 nucleotide substitutions **AAATAATT** in CroR-dependent regulatory motif TTTATTAA | This work |
| pSBT30 | pSBT25 with 6 nucleotide substitutions T**AATAAT**A in CroR-dependent regulatory motif TTTATTAA | This work |
| pSBT31 | pSBT25 with 4 nucleotide substitutions TT**ATAA**AA in CroR-dependent regulatory motif TTTATTAA | This work |
| pSBT38 | pJLL170 with 8 nucleotide substitutions **AAATAATT** in CroR-dependent regulatory motif TTTATTAA | This work |
| pJH086 | *E. faecalis* allelic exchange vector (Cm$^{r}$); *pheS** counterselection | 14 |
| pSBT40 | *pbp4(5)* promoter from OG1 with 4 nucleotide substitutions (ATAA) in pJH086 | This work |

| | | |
|---|---|---|
| pSBT64 | *pbp4(5)* promoter from CK221 with 4 nucleotide substitutions (ATAA) in pJH086 | This work |
| pJRG9 | *E. faecalis* expression vector with constitutive P23s promoter ($Cm^r$) | 2 |
| pJLL255 | *pbp4(5)* from OG1 in pJRG9 | This work |
| pCPN1 | *OG1RF_RS05560* in pJRG9 | This work |
| pJH123 | *E. faecalis* expression vector with constitutive P23s promoter ($Cm^r$) | 13 |
| pSLK245 | *OG1RF_RS05565-HA* with C-terminal HA tag in pJH123 | This work |
| pEAW9 | *pbpA(2b)* from OG1 in pJRG9 | 8 |
| pSLK239 | *OG1RF_RS11270-HA* with C-terminal HA tag in pJH123 | This work |
| pSLK238 | *OG1RF_RS05340-HA* with C-terminal HA tag in pJH123 | This work |
| pSLK237 | *OG1RF_RS03825-HA* with C-terminal HA tag in pJH123 | This work |
| pSLK241 | *OG1RF_RS07205-HA* with C-terminal HA tag in pJH123 | This work |
| pSLK243 | *OG1RF_RS03660-HA* with C-terminal HA tag in pJH123 | This work |
| pSLK244 | *OG1RF_RS05215-HA* with C-terminal HA tag in pJH123 | This work |
| pSLK250 | *OG1RF_RS12755-HA* with C-terminal HA tag in pJH123 | This work |
| pCJK245 | *E. faecalis* allelic exchange vector ($Cm^r$) | 15 |
| pSLB72 | Δ*OG1RF_RS05215* (ΔL7-T268, 95% deletion) in pCJK245 | This work |
| pSLB90 | Δ*OG1RF_RS02555* (ΔK7-D380, 96% deletion) in pCJK245 | This work |
| pSLK233 | Δ*OG1RF_RS12755* (ΔK7-K532, 97% deletion) in pJH086 | This work |
| pJLL286 | *E. faecalis* expression vector with $P_{nisA}$ nitrate-inducible promoter ($Em^r$) | 16 |
| pJLL310 | *pbpA(2b)* from OG1 in pJLL286 | This work |

## Supplemental references


1. Gold, O. G., Jordan, H. V. & van Houte, J. The prevalence of enterococci in the human mouth and their pathogenicity in animal models. *Arch. Oral Biol.* **20**, 473-IN15 (1975).

2. Snyder, H., Kellogg, S. L., Skarda, L. M., Little, J. L. & Kristich, C. J. Nutritional Control of Antibiotic Resistance via an Interface between the Phosphotransferase System and a Two-Component Signaling System. *Antimicrob. Agents Chemother.* **58**, 957–965 (2014).

3. Kellogg, S. L. & Kristich, C. J. Functional Dissection of the CroRS Two-Component System Required for Resistance to Cell Wall Stressors in Enterococcus faecalis. *J. Bacteriol.* **198**, 1326–1336 (2016).

4. Kristich, C. J. & Little, J. L. Mutations in the β Subunit of RNA Polymerase Alter Intrinsic Cephalosporin Resistance in Enterococci. *Antimicrob. Agents Chemother.* **56**, 2022 (2012).

5. Sahm, D. F. *et al.* In vitro susceptibility studies of vancomycin-resistant Enterococcus faecalis. *Antimicrob. Agents Chemother.* **33**, 1588 (1989).

6. Djorić, D. & Kristich, C. J. Extracellular SalB contributes to intrinsic cephalosporin resistance and cell envelope integrity in Enterococcus faecalis. *J. Bacteriol.* (2017) doi:10.1128/JB.00392-17.

7. Djorić, D. & Kristich, C. J. Oxidative Stress Enhances Cephalosporin Resistance of Enterococcus faecalis through Activation of a Two-Component Signaling System. *Antimicrob. Agents Chemother.* (2015) doi:10.1128/aac.03984-14.

8. Djorić, D., Little, J. L. & Kristich, C. J. Multiple low-reactivity class B penicillin-binding proteins are required for cephalosporin resistance in enterococci. *Antimicrob. Agents Chemother.* **64**, (2020).

9. Maekawa, S., Yoshioka, M. & Kumamoto, Y. Proposal of a New Scheme for the Serological Typing of Enterococcus faecalis Strains. *Microbiol. Immunol.* **36**, 671–681 (1992).

10. Kristich, C. J. *et al.* Development and Use of an Efficient System for Random mariner Transposon Mutagenesis To Identify Novel Genetic Determinants of Biofilm Formation in the Core Enterococcus faecalis Genome. *Appl. Environ. Microbiol.* **74**, 3377 (2008).

11. Hayes, F., Daly, C. & Fitzgerald, G. F. Identification of the Minimal Replicon of Lactococcus lactis subsp. lactis UC317 Plasmid pCI305. *Appl. Environ. Microbiol.* **56**, 202 (1990).

12. Kellogg, S. L. & Kristich, C. J. Convergence of PASTA kinase and two component signaling in response to cell wall stress in Enterococcus faecalis. *J. Bacteriol.* (2018) doi:10.1128/JB.00086-18.

13. Kristich, C. J., Little, J. L., Hall, C. L. & Hoff, J. S. Reciprocal Regulation of Cephalosporin Resistance in Enterococcus faecalis. *MBio* **2**, (2011).

14. Kellogg, S. L., Little, J. L., Hoff, J. S. & Kristich, C. J. Requirement of the CroRS two-component system for resistance to cell wall-targeting antimicrobials in Enterococcus faecium. *Antimicrob. Agents Chemother.* (2017) doi:10.1128/AAC.02461-16.

15. Kristich, C. J., Djoric, D. & Little, J. L. Genetic Basis for Vancomycin-Enhanced Cephalosporin Susceptibility in Vancomycin-Resistant Enterococci Revealed Using Counterselection with Dominant-Negative Thymidylate Synthase. (2014) doi:10.1128/AAC.02001-13.

16. Mascari, C. A., Djoric, D., Little, J. L. & Kristich, C. J. Use of an Interspecies Chimeric Receptor for Inducible Gene Expression Reveals that Metabolic Flux through the Peptidoglycan Biosynthesis Pathway is an Important Driver of Cephalosporin Resistance in Enterococcus faecalis. *J. Bacteriol.* **204**, (2022).
