# Supplementary material for: CroR Regulates Expression of pbp4(5) to Promote Cephalosporin Resistance in Enterococcus faecalis
Source: mBio. 2022 Aug 1;13(4):e01119-22. doi: 10.1128/mbio.01119-22 (PMC9426447; doi:10.1128/mbio.01119-22)
Supplement: TABLE S6 [file mbio.01119-22-s0009.pdf]

Supplemental Table 6. Primers used in this study.

Primer Name	5' - 3' sequence	Use	Source or reference
croR SP1 ST	CACCCGAGCCATTACTTCTAAAGGATTG	5'RACE	This work
croR SP2 ST	GACATAATCGTCTGCACCAGCAACTAAG	5'RACE	This work
croR SP3 ST	CCATATCGGTTGTTTTTGCTGTTAAC	5'RACE	This work
pbp5 SP1 ST	CATCACTAGCTTTGACTCCTTCTGC	5'RACE	This work
pbp5 SP2 ST	GAATCCCTGAATAAATTGCTTGG	5'RACE	This work
pbp5 SP3 ST	CTTTAAGCTAGCTTCTTGAACAACGGACGG	5'RACE	This work
ST RTqPCR 16S rRNA FWD	CAAGCGTTGTCCGGATTTATTG	qPCR	This work
ST RTqPCR 16S rRNA REV	GCACTCAAGTCTCCCAGTTT	qPCR	This work
SK pbp5_Set1_F	GTATCTTTTGTCGCAGCCCC	qPCR	This work
SK pbp5_Set1_R	GTGTTTGATGAAGTGGGCGT	qPCR	This work
